# A qualitative study exploring experiences of racial minority stress in pharmacy education and practice

**DOI:** 10.1016/j.rcsop.2024.100461

**Published:** 2024-06-07

**Authors:** Arisha Ahmed, Michael Hagos, Immer Bhatti, Nia Cartwright, Orieoma Chukwu-Etu, Angela Burini, Lola Dabiri, Clare Tolley, Charlotte Lucy Richardson, Amandeep Doll, Tanya Miah, Adam Pattison Rathbone

**Affiliations:** a Newcastle Pharmacy School, Newcastle University, Newcastle Upon Tyne, UK; b Royal Pharmaceutical Society, London, UK; c The Newcastle upon Tyne Hospitals NHS Foundation Trust, Newcastle Upon Tyne, UK; d United Kingdom Black Pharmacists Association, London, UK

**Keywords:** Racial minority stress, Pharmacist, Pharmacy, BAME, Healthcare, People of colour, Minority groups

## Abstract

**Background:**

Despite 49.1% of registered pharmacists in the UK being from a Black, Asian and Minority Ethnic (BAME) background, senior management roles within pharmacy are dominated by white males. People from BAME communities may experience minority stress which contributes to a professional attainment gap compared with non-BAME colleagues. Minority stress describes additional stressors, such as unconscious bias, micro-aggression and racial minority stress, experienced by minoritized people to adhere to the social norms of the majority. There is little evidence describing experiences of minority stress in pharmacy practice and education. The aim was to explore experiences of racial minority stress in pharmacy education and practice.

**Methods:**

A convenience sample of pharmacy students and pharmacists were recruited via email and social media posts to volunteer to take part in interviews and focus groups. A topic guide was used to explore experiences of unconscious bias, microaggressions and racial minority stress in education and practice. Interviews and focus groups were transcribed verbatim and inductively analysed using thematic analysis underpinned by a phenomenological approach. Ethical approval was granted from Newcastle University (5340/2020, 2430/2593).

**Results:**

Forty-five participants were recruited. Six focus groups and sixteen one-to-one semi-structured interviews were conducted. The sample was varied, with 56% (*n* = 25) students and 33% (*n* = 15) registered pharmacists from community, hospital, primary care, academia and an additional 11% (*n* = 5) still in foundation training in these sectors. The sample include diversity of racial identities, including 40% (*n* = 18) South Asian, 27% (*n* = 12) White, 15% (*n* = 7) Black, 7% (*n* = 3) Chinese and Arab mixed, 2% (n = 1) and 2% (n = 1) Not disclosed. Three themes were identified – Theme 1) Experiences of racial minority stress, Theme 2) Making sense of racial minority stress, and Theme 3) Responding to racial minority stress. Participants characteristics (for example skin colour, dialect, religious dress) made them feel susceptible to judgement, racist comments and microaggressions in education and practice. Participants required time to interpret, understand and make sense of incidents of racial minority stress. Responses to stressors included ‘ignoring ignorance’ and using a ‘professional identity’ to mask feelings. However, malicious comments and actions from other pharmacy staff were responded to differently to experiences from patients. Participants reported poor self-confidence to challenge racist behaviours in the workplace.

**Conclusions:**

The aim of this study was to explore experiences of racial minority stress in pharmacy education and practice. This study shows dealing with microaggression, racial minority stress and judgement in pharmacy education and practice is a burden experienced by people from BAME backgrounds. These experiences could contribute to the professional attainment gap in pharmacy, as making sense of these experiences is an additional burden pharmacists and trainees must bear in comparison to people from non-BAME backgrounds. Further work is needed to explore interventions to reduce minority stress in pharmacy practice and education to reduce the attainment gap across the sector.

## Highlights

• Little was known about experiences of racial minority stress in pharmacy education and practice.
• Micro-aggressions, unconcious bias and racism in pharmacy practice and education contributed to racial minority stress.
• Patients and professional colleagues contributed to racial minority stress in different ways.
• Interventions could be developed to support people experiencing racial minority stress.
• Experiences of racial minority stressors appeared to influence beliefs about professional attainment.

## 1 Background

Minority stress refers to stressors related to a minority status, such as race, ethnicity, culture or religion and is linked to stigma, prejudice, and discrimination in a hostile and stressful social environment, which can negatively influence mental health and well-being.^1^^,^^2^ These stressors can require time to cognitively process^3^ and lead to physiological changes.^4^ This harm may not be recognised or accounted for in practice and education settings. Racial minority stress can be differentiated according to the context it is experienced in and is typically dichotomised as malicious, ignorant, systemic or personal.^5^ For example, ignorance-based racial minority stressors refer to a lack of understanding about an individual's race, ethnic background, or religious beliefs. Malicious racial minority stressors describe behaviours with intention to cause harm to an individual based on their minority characteristics. Systemic racial minority stressors refer to the practices and policies of a place or organisation within society which is unfair or harmful to individuals or groups based on their race, ethnic background, or religious beliefs. Differentiating types of racial minority stress is important as it provides a structured approach to understanding experiences of racial minority stress and how this may inform experiences of minority stress more broadly.

Understanding minority stress has been linked to professional attainment. Professional attainment is influenced by socioeconomic factors linked to culture, race and ethnicity.6., 7., 8., 9., 10 This is particularly the case in large healthcare systems, such as the National Health Service (NHS) in the UK, which is one of the largest employers in the world.^11^ For example, although 20% of the NHS workforce identifies as being part of Black, Asian or Minority Ethnic (BAME) group, only 13% of senior managers identify themselves as BAME.^11^ The NHS Workforce Race Equality Standard (WRES) 2022 data analysis report showed that the percentage of Black and Minority Ethnic (BME) employees in the NHS was 24.2% but only 10.3% of senior positions were held by BME staff (WRES 2023).^12^ Negative experiences of racial minority stressors experienced in healthcare education and practice are well reported in the literature.^9^^,^^10^^,^^13^ A proposed hypothesis to the attainment gap in education and practice is that people from BAME backgrounds must work harder than their White counterparts due to the racial minority stress they experience in their everyday working lives. However, there is limited academic work exploring perspectives of racial minority stress in health education and practice, particularly in pharmacy.

Pharmacy education and practice represent a unique opportunity to understand racial minority stress in healthcare. Pharmacy education in Great Britain takes a minimum of five years and starts with an accredited four-year Master of Pharmacy degree followed by a 52-week foundation training placement and then registration with the General Pharmaceutical Council (GPhC). Once registered, pharmacists supervise the safe, effective and legal supply of medicinal products. The majority of registered pharmacists work in community or retail pharmacy settings, with the next largest cohort working in hospital and primary care settings and the remainder working across the pharmaceutical industry, academia, care homes and other settings.^14^ The attainment gap for women and people from ethnic minority backgrounds in pharmacy education and practice is also well recognised,^10^^,^^15^ with interventions to reduce the attainment gap reported in the literature.^16^ However, pharmacy represents a unique opportunity because approximately 49.1% of registered pharmacists in the UK identify as BAME, compared to just 15.4% of the population.^14^^,^^17^ This makes experiences of racial minority stress, professional attainment and racial disparities within the pharmacy sector more significant. As one of the few healthcare professions with increased representation of people from BAME backgrounds, the setting presents a unique opportunity to explore experiences of minority stress linked to race, ethnicity, religion and culture. This is because experiences of minority racial stressors are likely to influence more individuals in the sector overall, due to the high number of people from BAME backgrounds. There is potential then for a large proportion of the pharmacy sector to experience minority racial stressors, contributing to lower attainment for a large group of people in one sector. However, little is known about experiences of racial minority stress in pharmacy practice education or practice. The aim of this study was to explore experiences of racial minority stressors within pharmacy education and practice.

## 2 Methods

### 2.1 Study setting and design

The research team were based in North East England, which is a large geographical area with pockets of diversity situated within largely white, working class and middle class communities. The study used a qualitative approach underpinned by an interpretive phenomenological approach.^18^ The research team included experienced qualitative researchers (APR, CLR, CT), pharmacists (APR, CLR, CT, AD, TM), pharmacy students and trainees (IB, MH, AA) and a research assistant (NC). The study was conducted in phases; with phase one using focus groups to collect data and thematic analysis, phase two using interviews to collect data and thematic analysis and phase three to synthesise data from phase one and two using thematic synthesis. This approach enabled a diverse team to contribute to data collection and analysis. The research team included people from both BAME and non-BAME backgrounds. This was needed as racial minority stress is a sensitive topic and the diversity of the research team reduced the risk of bias in perspectives and interpretations of data and findings.

### 2.2 Participant recruitment and eligibility criteria

Participants had to be a current pharmacy student, foundation trainee or registered pharmacy professional, be based in the UK, and be able to consent to take part. Convenience sampling technique was used to recruit the sample through open digital advertisements (email, online posts) for volunteers for research in the research teams' professional networks, social media and word of mouth. It was not possible to record non-participation.

### 2.3 Data collection

One-to-one semi-structured interviews were conducted by one author (IB) and focus groups were conducted by two authors (MH, APR) online via Microsoft Teams with participants from across the UK between 1st October 2021 and 31st January 2023. Interviews and focus groups were chosen as they enable detailed, rich and insightful data about participants' lived experiences to be collected.^19^ All participants had access to information about the study and the opportunity to ask questions before agreeing to take part. Verbal consent was taken prior to starting data collection. Interviews and focus groups were digitally recorded and automatically transcribed using Microsoft Teams. Participants were signposted following the interview and focus groups to sources of support and help given the nature of the topic. A topic guide was used in both the interviews and focus groups which explored i) experiences of racial minority stress, ii) responding to racial minority stress and iii) the impact of racial minority stress on career development. Field notes were made during interviews and focus groups. Transcripts were quality checked by at least two authors) by listening to the recording, reading the transcript simultaneously, reading field notes and correcting errors. Inaccuracies were discussed until consensus to agree an amendment. Transcripts were not to participants for checking. All data recordings, transcripts and study documents were stored in password protected server with multifactor authentication and only accessible by the research team. No repeat interviews were carried out.

### 2.4 Data analysis

In phase one and two, data from interviews and focus groups were analysed independently by two teams (focus groups - APR, MH, interviews – CLR, IB) using thematic analysis. Thematic analysis uses a step-wise approach to familiarise researchers with the data, identify codes, clusters and themes to describe the findings.^20^ Interviews and focus groups were conducted until theoretical was reached. Data saturation is the point in the research process when no new information is discovered in data analysis.^21^ This reasonably assures that further data collection would yield similar results and serve to confirm emerging themes and conclusions.^21^ Data from interviews and focus groups were then analysed together in phase three using thematic analysis by two authors (AA, NC).^22^^,^^23^ A combination of manual and electronic coding in NVivo Version 1.2 was used, with an audit trail and codebook to ensure inter-coder reliability. Transcripts were read line by line and coding using an inductive, constant comparison approach to identify codes, clusters, and themes. Findings were shared with the research team (APR, CLR, AD, TM, CT) for interrogation to validate the findings. Data extracts were chosen by three authors (AA, CLR, APR) to be included in the manuscript to represent the voices of the participants. Participants were not invited to review the findings.

### 2.5 Ethical approval

Ethical approval was granted by the Faculty of Medical Sciences Ethics Committee in October 2020 (ref: 5340/2020) and in 2022 (ref: 2430/2593) to extend the end date of the study.

## 3 Results

Participants were recruited from a wide range of backgrounds (See Table 1) and included a mixture of sexes (male [25%, *n* = 11], female, [73%, *n* = 33] and undisclosed [2%, n = 1). The majority of participants were pharmacy students (56%, *n* = 25) with the remaining registered pharmacists or foundation training pharmacists working in foundation training [11%, *n* = 5], in academia and community [9%, n = 11] respectively, hospital [7%, n = 3], mental health [4%, n = 2] and national policy and primary care [2%, n = 1, respectively]). Participants also identified themselves as from a range of cultural, ethnic and racial backgrounds (South Asian [40%, *n* = 18], White [27%, *n* = 12], Black [15%, *n* = 7], Chinese and Arab [7%, n = 3, respectively], Mixed [2%, n = 1] and not disclosed 2%, n = 1]. Participants confirmed they were working or studying in the UK however no regional or locality data was collected. Interviews lasted an average of 30 min 20 s, and focus groups lasted on average 65 min 17 s. Themes are described below and supported by data extracts (See Table 2) which demonstrate participants experiences in their own words. Parts of the extracts are underlined by the authors to add emphasis. Participants are referred to by anonymous numbering along with contextual demographic information.

**Table 1:** Summary of Participant Demographics.

	n	%
Total	46	100%
Sex		
Female	33	72%
Male	11	24%
Not disclosed	2	4%

Self-identified race		
Arab	3	7%
Black	7	15%
Chinese	3	7%
Mixed Bengali/White	1	2%
South Asian	17	37%
White	13	28%
Not disclosed	2	4%

Sector		
Academia	4	9%
Community	4	9%
Foundation trainee pharmacist in community	1	2%
Foundation trainee pharmacist in GP practice	1	2%
Foundation trainee pharmacist in hospital	3	7%
Hospital	3	7%
Mental health	2	4%
National policy	1	2%
Primary care	1	2%
Student	25	54%
Not disclosed	1	2%

**Table 2:** Summary of themes identified following thematic analysis of interview and focus group data relating to racial minority stress in pharmacy practice and education.

Theme name	Theme description	Sub-themes
Experiences of racial minority stress	Many definitions of racial minority stress, unconscious bias, microaggressions and macro-aggressions were reported. All experiences appeared linked to racial, ethnic, cultural identity. Experiences linked to dialect influenced rapport building with colleagues and patients.	What is racial minority stress?
		Protected characteristics
		The role of dialect and racial minority stress
Making sense of racial minority stress	Participants struggling to identify racial minority stress as unconscious bias, microaggression or over racial minority stress (macro-aggression). Participants struggled to identify the ‘root of racial minority stress’ as ignorance or maliciousness but based this on expectations of racial minority stress. Participants expected healthcare professionals to have completed training and so know the harmful impact of racial minority stress. Patients were expected to be less educated, with racial minority stress rooted in ignorance rather than malice.	Was that racial minority stress?
		Ignorance from patients
		Maliciousness from staff
Responding to racial minority stress	Once participants understood racist comments, participants reported masking their racial, cultural and ethnic identity with a professional identity. Poor confidence in challenging racial minority stress due to fear of the consequences associated with speaking up. Conforming to normalised professional standards of being polite and courteous reduced motivation to report racial minority stress. Poor aspirational attainment for people from BAME backgrounds due to limited representation of people from BAME backgrounds in senior pharmacy roles.	Using professional identity to cope
		Lack of confidence
		Fear of consequences when bringing up issues with staff
		Professional standards prevent reporting
		No senior management roles

### 3.1 Theme 1: Experiences of racial minority stress

Participants described different definitions of racial minority stress which were unique to each participant but shared common elements. This included descriptions of protected characteristics and indicators of difference from the White majority ethnicity, race or cultural norm, such as accent, skin colour and religion. When asked what they believed racial minority stress was pharmacy staff and students described racial minority stress was categorised into different types, either ignorant or malicious acts and behaviours, or systemic cultures.

*“It could be anything from an ignorant comment or a direct comment that's malicious… It's a very broad term and it's hard to describe because there's structural racism… It could be structurally, and organisationally, not catering for a certain group of people.” – Participant 27, female, Chinese, hospital pharmacist.*

*“Racism is when there's an intent to be malicious and cause harm based on the individuals' backgrounds or race or ethnicity and it's used to overpower somebody. It's done to kind of put someone in their place or make someone feel inferior is how I would understand racial minority stress” – Participant 37, female, South Asian, policy maker.*

Participants went on to talk about their perception of microaggressions in education, such as when educators highlight differences in prescribing for minority populations, preferentially spend time with specific groups of students, timetable learning without consideration of culturally significant events (such as Ramadan and Holi), and teach using anecdotes, humour and jokes, which are culturally specific (and so not understood by people from other cultures). Participants reported this raised the feeling that actions or comments were deliberately subtle, which was used as a defence against being accused of racism in the future, or that they were “just joking”.

*“Sometimes [colleagues] know that they're doing it, but they do it in such a way that they can't be brought up on it again. There's a bit of a fail-safe where they could say, oh, I was just joking or oh, I didn't realize that was offensive’ – Participant 5, female, South Asian, pharmacy student).*

*“You almost get this feeling that there's racism underlying people's actions but if you were to try and prove that – you can't… You get that feeling, but you will never be able to prove it to anyone else.” – Participant 36, female, Black, pharmacy student.*

Participants from different sexual, gendered, racial, ethnic and cultural backgrounds described characteristics they believed would expose someone to experience racial minority stress.

*“I always say the trifecta of prejudice in this world, unfortunately. Black Muslim woman.” – Participant 21, male, Black, community pharmacist.*

*“I think it would be harder for a Muslim woman to get into [a senior] position” -Participant 34, female, South Asian, mental health pharmacist.*

*‘There's a lot of the pharmacy students of Asian background and a lot of them are female as well as being Asian. […] In places I've worked at I notice, and it tends to be males, not always, but it does tend to be white people that can really talk down to people. And yes, you notice that it's subtle. It's there, it's constant and it must be difficult” – Participant 17, male, White, community pharmacist.*

Most participants believed accent was an indicator to receiving racist comments from patients', due to a lack of confidence in the participants' professional's ability. Participants believed that patients would have concerns that the individual was poorly educated, making assumptions that non-white people had poorer quality education and consequently were less competent to practice as a healthcare professional.

*“The chance of patients not wanting to speak to [students with a minority accent] is higher and purely because they don't think they have a clue what they're talking about… the patients might think that… they can't read, they can't write. There are so many misconceptions. So, I think, and I do feel sorry for, students who have accents because I do think that their chances of patients rejecting them [...] is higher.” Participant 6, female, South Asian, pharmacy student.*

*“A patient was questioning her [Indian] accent, questioning where she's from and is she actually the right person to be receiving advice from?’ – Participant 15, male, South Asian, pharmacy student.*

*“I do believe that some patients might be reluctant to deal with pharmacists who might have an accent or speak slightly different to them”’ – Participant 35, female, Arab, pharmacy student.*

Some participants believed it was because the patient did not feel comfortable with unfamiliar accents, due to concerns about being able to understand the healthcare professional. Using familiar phrases, terminology and shared social norms were important to build rapport. Participants believes patients were more open to discuss their health concerns if the healthcare professional had a familiar accent.

*“The patient was like, oh, can you say it because I can understand your accent better, so I guess mine's quite a local accent, so it may just be easier to understand. I don't think she was trying to be nasty.’ – Participant 3, female, South Asian, pharmacy student.*

*‘When I've been with people who have that regional […] accent, it's almost easier for them to get along with patients. And I find sometimes, my accent is obviously from [a different region]. And they're like, it's a bit harder to build up that kind of rapport with the patient or even clinical staff.’ – Participant 5, female, South Asian, pharmacy student.*

Participants reported that having a different regional accent from within the UK (e.g., Geordie or Scouse) uniquely compared to having an international accent (e.g., Indian). This appeared to be mediated by patient and professional racial and cultural biases.

*“I think it comes down to people's patience and racial biases. For example, I feel like if someone had say a Liverpool thick Scouse accent as opposed to an international accent. Both different to the dialect and accents… but people would have a completely different reaction in understanding a Scouse accent compared to an international accent… They would come to that situation with like a different attitude and I think it all comes down to their, like, racial biases.” – Participant 13, male, White, pharmacy student.*

Experiences of having a minority accent appeared to influence perceptions of education, competence and attainment in pharmacy practice and education. This made building rapport with patients challenging. For individuals with international accents who also identified as BAME, participants felt this was an extra hurdle that their native English-speaking counterparts may not struggle with, thus creating an inequality in practice and education.

### 3.2 Theme 2: Making sense of racial minority stress

Participants were unsure if their negative social experiences in pharmacy education and practice were inappropriate or wrong and if so, rooted in unconscious bias, prejudice, racial microaggression or overt (macro-aggressive) racial minority stress. Overt racial minority stress refers here to experiences which were considered very focused intentional and deliberate behaviours or speech whereas unconscious bias, prejudice and microaggressions were considered unintentional and general. Specific time and discussion were required to understand if a comment was racist or not, and what action was needed and would, or should, be taken in response to the exposure.

*“I think a micro aggression is something that's kind of hard to pinpoint If it was that. So, I think talking to your friends, especially friends who have the same characteristics, and them saying that ‘that's definitely something bad that was said’ helps you to understand.” – Participant 2, female, South Asian, pharmacy student.*

*“I kind of ignored it and then I was just like, what just happened? I just swept it under the rug [because] I wasn't sure” – Participant 5, female, South Asian, pharmacy student.*

*“I think with microaggressions it takes time to process what has happened and it can really affect someone's mental health.” – Participant 13, male, White, pharmacy student.*

*“I feel sometimes uncomfortable because I'm like am I saying the right thing? But then sometimes I'm thinking well, I'm pretty inexperienced so maybe it's just I need to learn. But how much of that is the racial backgrounding, and how much of it is inexperience? It's difficult to say” -Participant 33, male, South Asian, community pharmacist.*

Participants described understanding racist behaviours from patients as typically understood as rooted in ignorance but behaviours from healthcare professionals as malicious. This appeared to be because of an expectation that healthcare professionals had had equality, diversity and inclusivity training, had professional practice standards and exposure to progressive norms which should reduce racist behaviours. Therefore, healthcare professionals were expected to know racist behaviours were intended to be detrimental, discourteous and derogatory.

*“I think patients would be more inclined to be micro aggressive than hospital staff. I think if you're in a medical field, you're more experienced with diversity through teaching with people you have studied with or patients coming from all different backgrounds. You kind of have to adapt to know how to treat everyone fairly, treat everyone with respect”’ Participant 1, female, South Asian, pharmacy student.*

*“Maybe [patients are] not as educated and maybe sometimes, are not as respectful. Other times they may have been waiting for a very long time for hospital staff so, they get frustrated, and say things that they don't mean” – Participant 2, female, South Asian, pharmacy student.*

This extended to healthcare professionals who held senior positions in pharmacy practice, where decision-making was considered racist as it did not consider the safety needs of people from BAME backgrounds to the same extent as non-BAME colleagues.

*“we know several studies published [about COVID] say BAME people get more severe symptoms and deteriorate when they get it. So then when the second wave comes along and then [senior managers] want to restart opening Covid wards. Why do they want one of the BAME pharmacists to cover it when there's like 20 or 30 other White British pharmacists that could cover it that day.” – Participant 27, female, Chinese, hospital pharmacist.*

*“I was working in a particular area of the hospital [managing patients with COVID] once in training and no one seemed to bat an eyelid that I was there on the frontline but when it was my counterparts, [who is an older white male], turn to be on that same ward, everyone was like ‘Oh my God no you can't go there! It's not safe for you’ … why did no one say anything or even mention anything when I was there?” – Participant 21, male, Black, community pharmacist.*

In summary, the participants experienced racial minority stress as microaggressions, unconscious bias, and overt racism (macro-aggressions) which made it difficult to decipher if the comments and actions were rooted in ignorance or malice. It took time to process, understand, and make sense of these experiences which helped through reflection and discussion with trusted peers.

### 3.3 Theme 3: Responding to racial minority stressors

Participants described responding to racist experiences in pharmacy practice and education in different ways (either ignoring it or taking action), which was mediated by their beliefs about the root of behaviours. For experiences with patients, participants felt they had to maintain a professional identity, and conform to professional standards, to prevent an emotional response impacting the way they provided care for patients. This manifested as not responding, challenging or ‘speaking up’ about their experiences.

*“[after a patient has said something racist] I'd try to be more polite to show that I am a normal person” – Participant 1, female, South Asian, pharmacy student.*

This appeared more pertinent for those earlier in their careers who described that fears about the impact speaking up may have on training and career progression in pharmacy education.

*“[being seen to be] arguing with the patient is not very appropriate […] We are only students, I think that's the reason why I didn't say anything” -Participant 4, male, undisclosed race, pharmacy student.*

*“if someone sees you [playing the race card] as a student, they're not going to take you as seriously as a healthcare professional [so] I can't say anything. So, l just stayed calm and tried to stay professional” – Participant 14, female, Arab, pharmacy student).*

This sentiment was echoed by participants in practice and education who also reported ‘downplaying’ experiences of racism to avoid being labelled as ‘troublesome’ and slowing down their career progression.

*“I don't want to be negatively judged or hindered because I've raised [objection to a micro aggression] which may or may not be racist”- Participant 40, female, White, pharmacy student.*

*“It's just a thing of being seen as like being difficult and by complaining and things like that […] You feel like you might be compromising a future job position and things like that”- Participant 45, female, South Asian, foundation trainee pharmacist working in hospital.*

Additionally, poor representation of non-White people in senior roles in pharmacy practice and education reinforced beliefs high professional attainment was linked to race, ethnic and cultural identity.

*“I think it can affect promotions or progression maybe? [...] You don't see as many BAME pharmacists within high positions and stuff such as that”- Participant 41, female, South Asian, foundation trainee pharmacist working in hospital.*

This belief was echoed by White participants.

*“Many [BAME] friends have said to me - I'm brown, I know I can't go higher than this level” – Participant 23, undisclosed sex, White, primary care pharmacist.*

Differences in attainment between White and non-White pharmacists were reported as a culturally normal phenomenon, with participants indicating this was a routine, commonly accepted understanding that non-White pharmacists had lower professional attainment than their White colleagues.

Participants described responding passively to micro aggressive, racial behaviours partly because of their interpretation of the role of professional standards in education and practice, which pharmacists need to be polite and courteous, or ‘conform to the norm’, in order to progress their training and career in practice.

*“I think I handled it well because, if I wasn't in a professional environment, I'm telling you I would have been coming a very different way, but of course there were people there and I had to keep my cool.” – Participant 16, female, South Asian, pharmacy student.*

*‘That is always a fear that I have. I feel especially if [racism is] coming from someone in a manager role or higher up and your career is in their hands. They are the one that are going to represent you. They are the ones that are going to recommend you and at the end of the day pharmacy isn't as big as is it.” – Participant 35, female, Arab, pharmacy student.*

*“a lot of my friends […] couldn't raise their concerns because they were really worried that their manager wouldn't pass them on their competencies, or they wouldn't be given support. Which has happened to some of them. They had to retake the exam and failed the exam”’ – Participant 23, undisclosed sex, white, primary care pharmacist.*

One participant spoke about how they even tried to leave their job due to ‘a racist culture.’

*“They said ‘they've got a lot of [foundation training pharmacists] and pharmacy students from an ethnic minority background, and we don't want too many.” They actually said that in front of me and at that point I didn't feel confident enough to be able to challenge it. […] Insidious type of comments that you're not sure if they are racist or not. You know that they are, but it's really hard to call that out. So, I really didn't like working there and I got out as soon as I could.” – Participant 34, female, South Asian, mental health pharmacist.*

Additionally, the routine nature of exposure to racial minority stressors meant participants became desensitised, tolerant and passive to racist behaviours.

*“obviously there's a zero tolerance here for that kind of stuff, but you do end up almost being desensitised to it because it's just routine if that makes sense” – Participant 34, female, South Asian, mental health pharmacist.*

To summarise, responses to racial minority stressors appeared to be passively accepting, rather than actively challenging the behaviour, and mediated by beliefs that ‘conforming to the norm’ set by professional standards would enable continued progression in pharmacy education and practice. Participants from BAME and non-BAME backgrounds believed attainment was limited by minority racial, ethnic and cultural identities.

## 4 Discussion

This is the first study to describe the lived experiences of racial minority stress in pharmacy education and practice. Trainee and registered pharmacists felt judged based on their characteristics such as accent, race, ethnicity and cultural identity, manifesting experiences of racial minority stress as unconscious bias, microaggressions and overt macro-aggressive racism from both patients and colleagues. Pharmacists and trainees needed time and energy to make sense of these experiences, to identify what happened and how to respond with difficulty interpreting the root of the behaviours. Findings identified pharmacists and trainees interpreted racial minority stressors differently depending on if it came from patients or other professionals however racial minority stressors were passively accepted regardless. Both trainees and registered pharmacists reported few experiences of speaking up to actively challenge racial minority stressors due to a fear of repercussions on their own training and career progression. Limited representation of senior leaders from BAME backgrounds meant aspirational attainment of pharmacists and trainee from BAME was described to be lower than their non-BAME counterparts. These experiences collectively demonstrate experiences of racial minority stress in pharmacy practice and education whereby experiencing, interpreting and responding to racism contributes to stress and influences attainment.

Understanding racial minority stress in pharmacy practice and education in the UK is important as pharmacists are a cornerstone of healthcare in an increasingly diverse population. The nature of the pharmacy profession is diverse in comparison to the general population and so represents a unique opportunity to understand experiences of minority stress. Experiences of minority stress in the workplace can contribute to poor mental health, reduced productivity and performance and professional attainment.^1^^,^^2^^,^9., 10, 11.^,^^15^ As almost half of pharmacists are from BAME backgrounds,^14^ this means almost half of the workforce may experience racial minority stress contributing to limited career progression for a large proportion of the workforce. This is further backed by the Pharmacy Workforce Race Equality Standard data analysis report which states that in 2021 the percentage of BME staff that believed that their trust provides equal opportunities for career progression and promotion was 46.3% which is much lower than the 61.8% of white counterparts that believed the trust provided equal opportunity.^24^

This is important as pharmacists are increasingly called upon to deliver front-line health services in the NHS,^12^^,^24., 25., 26. as well as adopt senior leadership roles with strategic services. However, existing policy does not appear to consider the impact of racial minority stress on the pharmacy workforce or provide opportunities to account, recognise and reward the difficulties that up to half of registered pharmacists may deal with in their everyday working lives. The workforce strategy then must recognise that racial minority stress is a significant experience in the pharmacy sector and must account for this in policy, practice and education.

### 4.1 Limitations

This research uses a robust set of data with participants ranging in experience, sector, background and geographical areas of pharmacy. From a reflexive perspective, the were based in North East England and included experienced qualitative researchers with doctoral qualifications (APR, CLR, CT), pharmacists (APR, CLR, CT, AD, TM, OCE, AB, LD), pharmacy students and trainees (IB, MH, AA) and research assistants (NC). The authors include a diverse group reflecting a variety of sexual, gender, sexuality, ethnic, racial and culturally diverse identities. This reduced the risk of bias in the study. However, the findings may be limited in that data was collected in phases by two separate authors and then thematically analysed by a third author, with some discrepancies between self-identified ethnicity, race and cultural identity, which could not be clarified, and so only generic race data is reported here. Additionally, some researchers (APR, CLR, CT, AA, IB) and participants had existing relationships with some participants through involvement in pharmacy education, as either peers (AA, IB, MH) or former students (APR, CLR, CT). Some participants may therefore had had some awareness of the researchers' identities and perspectives about the goals of the research. Research team characteristics (if not already obvious like race, gender, culture) were discussed as part of the interviews and focus groups. This is problematic as it reduces the quality of the data by over-simplifying a complex and nuanced phenomenon (for example, participants identified above as ‘Black’, also identified ethnically as African or Caribbean and culturally as Ghanian, Nigerian, Jamaican). However, inconsistencies in the recording of data between the interview and focus group study meant inferences could not be made beyond racial identity. Due to the nature of qualitative research and convenience sampling used to recruit participants, findings are not generalisable to other settings. However, the findings may be transferable to similar contexts.^27^ A stratified sampling method could capture key population characteristics in the sample and improve the generalisability of the study.

Another limitation to this study is that the study relied on the accurate recall of the experiences the participants spoke about. Participants can misinterpret situations and incorrectly recall situations as the ability to recall accurate information reduces over time.^28^ Additionally, although the diversity of the research team may have provided a suitable, safe and welcoming space for participants to share their experiences, the data collected may be limited by the Hawthorne effect. This describes a phenomenon whereby research participants alter their behaviour or accounts in response to being observed.^29^ This is particularly problematic given the sensitive nature of racial minority stress, as non-White participants may not have wanted to share experiences whilst observed by White researchers or participants.^30^ Finally, it should also be recognised that no pharmacy owners participated in the study, only employees, trainees and students and so the findings may not reflect experiences of the practice of pharmacy owners.

Although this research has shown racial minority stress are significant experiences in pharmacy practice and education, the scale of these experiences has not yet been identified. Further research is needed to provide incidence and prevalence data relating to experiences of racial minority stress and support people who experience all forms of racism from professional colleagues and patients to appropriate authorities. Additional attention is also needed to support trainees and pharmacists to identify, understand and respond to experiences of racial minority stress from patients and identify ways to cope with, and manage, the additional workload that this will create for people who experience racial minority stressors in their everyday working lives. This is particularly important during periods of political turmoil such as elections or referenda, were issues such as migration, religion, and race, can become politicised and lead to changes in public sentiment to characteristics like accent, skin colour and religion. Finally, although this research identified beliefs that people from BAME backgrounds have limited opportunities for career progression and attainment, further work by professional bodies and senior leadership teams is needed to support these trainees and pharmacists to build aspirational career trajectories to develop the senior pharmacy leaders of the future.

Increasing the awareness of what racial minority stressors are, how they are experienced and what practitioners and educators can do to support people who experience them, is needed. Initiatives which increase awareness may go some way to reduce racial minority stressors, however limited examples of these initiatives exist. One example is the Inclusive Pharmacy Practice (IPP) initiative in the UK, which was launched by the Royal Pharmaceutical Society and Association of Pharmacy Technicians UK and 13 other collaborators.^26^ The initiative seeks to support organisations to adopt four principles, centred on leadership and representation (two principles), education and training (one principle) and healthcare service delivery (one principle). These ambitious principles focus on racial equality and cultural competence appear to be the first of their kind to specifically target improving experiences of pharmacy professionals in relation to work-place racial minority stressors. Existing resources to improve cultural competence are available,^31^ which provides training and signposting to other resources. Although novel, these initiatives may be over generalised away from specific concepts, such as racial minority stressors, to broader constructs, such as cultural competence. Evidence suggests cultural competence is poorly defined with limited integration into pharmacy education.^32^ Specific, objective and measurable standards are needed to provide structure and accountability for practitioners and educators. Although these initiatives are a step forward, further work is needed by global organisations and professional leadership bodies to recognise and reduce experiences of racial minority stressors in pharmacy practice and education.

## 5 Conclusion

This study aimed to explore racial minority stress in pharmacy practice and education. The findings demonstrate a wide variety of experiences of unconscious bias, microaggressions and overt racist macro-aggression. Making sense of experiences of racial minority stress took time and effort. Experiences of racial minority stressors from patients were interpreted differently to experiences with professional colleagues, who were expected not to be racist or contribute to racial minority stress. Responses to racial minority stressors were passive, with pharmacists and trainees fearful of repercussions on their career trajectory if they did not appear to ‘conform to the norm’ and meet professional standards for being polite and courteous by challenging racist behaviours. Further work must recognise the significance of experiences of racial minority stress on professional attainment, workforce productivity and career progression.

## Funding

No funding was received to support this project.

## CRediT authorship contribution statement

**Arisha Ahmed:** Writing – review & editing, Writing – original draft, Formal analysis. **Michael Hagos:** Writing – review & editing, Project administration, Methodology, Investigation, Formal analysis, Data curation. **Immer Bhatti:** Writing – review & editing, Project administration, Methodology, Investigation, Formal analysis, Data curation. **Nia Cartwright:** Writing – review & editing. **Orieoma Chukwu-Etu:** Writing – review & editing. **Angela Burini:** Writing – review & editing. **Lola Dabiri:** Writing – review & editing. **Clare Tolley:** Writing – review & editing, Writing – original draft, Supervision, Project administration, Formal analysis. **Charlotte Lucy Richardson:** Writing – review & editing, Writing – original draft, Visualization, Validation, Supervision, Methodology, Investigation, Formal analysis, Data curation, Conceptualization. **Amandeep Doll:** Writing – review & editing, Writing – original draft, Validation, Supervision, Conceptualization. **Tanya Miah:** Writing – review & editing, Writing – original draft, Supervision, Methodology, Formal analysis, Conceptualization. **Adam Pattison Rathbone:** Writing – review & editing, Writing – original draft, Visualization, Validation, Supervision, Software, Resources, Project administration, Methodology, Investigation, Funding acquisition, Formal analysis, Data curation, Conceptualization.

## Declaration of competing interest

The authors declare they have no known competing financial interests or personal relationships that could have appeared to influence the work other than what is already reported in this paper.

## Data Availability

Anonymised data is available from the corresponding author upon reasonable request, subject to agreement.

## Appendix A Supplementary data

Supplementary material 1 COREQ Checklist Image 2

## References

[1] Wong C.F. (2014). Minority stress experiences and psychological well-being: the impact of support from and connection to social networks within the Los Angeles house and ball communities. Prev Sci.

[2] Meyer I.H. (2003). Prejudice, social stress, and mental health in lesbian, gay, and bisexual populations: conceptual issues and research evidence. Psychol Bull.

[3] Ozier E.M., Taylor V.J., Murphy M.C. (2019). The cognitive effects of experiencing and observing subtle racial discrimination. Aust J Soc Issues.

[4] Harrell J.P., Hall S., Taliaferro J. (2003). Physiological responses to racism and discrimination: an assessment of the evidence. Am J Public Health.

[5] Ryan J. (2003). Educational administrators' perceptions of racism in diverse school contexts. Race Ethn Educ.

[6] Fouad N.A., Kantamneni N. (2013). The role of race and ethnicity in career choice, development, and adjustment. Career Dev Couns Theory Res Work.

[7] Lee J., Zhou M. (2014). The success frame and achievement paradox: the costs and consequences for Asian Americans. Race Soc Probl.

[8] Adhikari R. (2023). It's ok to be different: supporting black and minority ethnic nurses and midwives in their professional development in the UK. Nurse Educ Pract.

[9] Kar P. (2023). Partha Kar: if you won't speak up, how will the world know you exist?. BMJ.

[10] Johnston L., Cameron G., Vanson T. (2016). Qualitative research into the registration assessment performance among black african candidates. OPM.

[11] Chasma F., Khonat Z. (2021). How equal is access to senior management and leadership roles in the NHS?. Br J Healthc Manag.

[12] England N.H.S. (2022). NHS Workforce Race Equality Standards (WRES). https://www.england.nhs.uk/long-read/nhs-workforce-race-equality-standard-wres2022-data-analysis-report-for-nhs-trusts/.

[13] Frings D., Gleibs I.H., Ridley A.M. (2019). What moderates the attainment gap? The effects of social identity incompatibility and practical incompatibility on the performance of students who are or are not black, Asian or minority ethnic. Soc Psychol Educ.

[14] General Pharmaceutical Council (2022).

[15] Connelly D., Burns C. (2023). Infographic: the ethnicity awarding gap for the pharmacy degree during COVID-19. The Pharma J, PJ.

[16] Lunn A., Manfrin A. (2023). Impact of reduced idea density on pharmacy students' attainment in pharmaceutical calculations: a study protocol for a single-blind multicentre randomised controlled trial. Int J Educ Res.

[17] Statistics, O.f.N., GOV.UK Ethnicity facts and figures. 2023.

[18] Tuohy D. (2013). An overview of interpretive phenomenology as a research methodology. Nurse Res.

[19] Crotty M.J. (1998). The foundations of social research: meaning and perspective in the research process. The Foundations of Social Res.

[20] Clarke V., Braun V., Hayfield N. (2015).

[21] Faulkner Sandra L., S.P.T (2017). Data saturation. The international encyclopedia of communication research. Methods.

[22] Edgar T.W., M.D (2017). Exploratory study. Research methods for cyber. Security.

[23] Baran M., Jones J. (2016).

[24] NHS England (2023). Pharmacy Workfore Race Equality Standard report. https://www.england.nhs.uk/long-read/pharmacy-workforce-race-equality-standard-report/#:~:text=Black%2C%20Asian%20and%20minority%20ethnic%20female%20(14.2%25)%20and%20Black,6.1%25)%20and%20White%20male%20.

[25] NHS England (2020). NHS Long Term Plan. https://www.longtermplan.nhs.uk/.

[26] NHS England (2023).

[27] Straus S.E. (2013). Characteristics of successful and failed mentoring relationships: a qualitative study across two academic health centers. Acad Med.

[28] Stanley S.E., Benjamin A.S. (2016). That's not what you said the first time: a theoretical account of the relationship between consistency and accuracy of recall. Cognitive Research: Principles and Implications.

[29] Jones S.R. (1992). Was there a Hawthorne effect?. Am J Sociol.

[30] Bergerson A.A. (2003). Critical race theory and white racism: is there room for white scholars in fighting racism in education?. Int J Qual Stud Educ.

[31] Baqir W., Root G. (2021).

[32] Jarrar R. (2023). How cultural competence is conceptualised, developed and delivered in pharmacy education: a systematic review. Int J Clin Pharmacol.

